# Sulfated endospermic nanocellulose crystals prevent the transmission of SARS-CoV-2 and HIV-1

**DOI:** 10.21203/rs.3.rs-2163527/v1

**Published:** 2022-10-28

**Authors:** Enrique Javier Carvajal Barriga, Wendy Fitzgerald, Emilios K. Dimitriadis, Leonid Margolis, R. Douglas Fields

## Abstract

Biomaterials with antimicrobial activity are gaining attention due to their biodegradability and efficacy in interacting with a wide range of microorganisms. A new cellulose nano-biomaterial, endospermic nanocellulose crystals (ENC) obtained from parenchymal tissue of ivory nut endosperm, has a natural capacity as a universal binder. This feature is enhanced when it is chemically functionalized, and can be exploited in the fight against microbes. We tested the ability of sulfated ENC in aqueous suspension to encapsulate viruses through a crosslinking reaction mediated by cations. 0.25% w/v ENC suspensions efficiently encapsulated spike (S) protein, preventing its interaction with ACE2 receptor. ENC was further able to encapsulate SARS-CoV-2 pseudoviruses and prevent infection of 293T-ACE2 cells. ENC also suppressed infection of MT-4 cells with HIV-1 _LAI.04_ . This antiviral activity of sulfated ENC is due to the irreversible interaction of ENC with viral particles mediated by crosslinking, as antiviral activity was less effective in the absence of cations. Additionally, ENC was used as a matrix to immobilize recombinant ACE2 receptors and anti-S IgG, creating molecular lures that efficiently inhibited SARS-CoV-2 infections *in vitro* . These results show that sulfated ENC from ivory nuts can be used as an efficient antiviral material.

## Introduction

The primary mode of SARS-CoV-2 transmission is by inhalation of respiratory droplets, thus the use of masks is recommended to limit transmission. Numerous human studies have shown that masking reduced transmission[1]. Air filtration by HEPA filters also reduced exposure up to 65–90%[2]. One method to further reduce viral transmission would be to develop materials that combine filtration with antimicrobial properties.

Towards this goal we proposed the use of cellulose nanoparticles to encapsulate viral particles thus preventing their passage. These nanocellulose particles were designed with dense sulfate charges obtained from ivory nuts from a palm tree from the *Arecaceae* family that grows in tropical regions of South America. Due to its derivation from the endosperm of the palm´s seed, it was designated endospermic nanocellulose (ENC). The unique primary cell walls yielded more homogeneous and thinner cellulose nanoparticles compared to cell walls from other vegetal tissues. The thinner the nanoparticles, the higher the surface area of the suspended material to interact with the viruses. This material could be used as a film for masks and filters to bind viral particles, increasing the effectiveness of these filtration devices.

Here we demonstrated two different methods of blocking transmission of virus using sulfonated ENC. One was non-specific that consisted of the direct binding (nanoencapsulation) of the SARS-CoV-2 pseudovirus particles, or encapsulation of 293T-ACE2 target cells. ENC also efficiently encapsulated HIV-1 virions and prevented transmission to MT-4 target cells. The second method was highly specific using molecular lures, anti-SARS-CoV-2 spike (S) protein antibodies and soluble ACE2 receptors, immobilized in nanocellulose scaffolding to specifically bind viral particles.

Antiviral activity of specific biomaterials depends on different modes of action such as the interference with virus entry in cells; the destruction of viral structures; the inhibition of replication of nucleic acids; and inhibition of virus release from infected cells[3,4]. Sodium alginate, a soluble salt of alginic acid that crosslinks in presence of $Ca^{++}$ ions, has been tested with successful results against several viruses, including SARS-CoV-2 and HIV-1. The antiviral mechanisms include anionic interaction of alginic acid with viral surface proteins that interferes with viral receptor interactions, inhibition of capsid synthesis, and the aggregation and encapsulation of viral particles which limits their infectivity[5]. Recently, sialic acid has also been found to bind to a specific domain in the S protein of SARS-CoV-2, which can be exploited to inhibit SARS-CoV-2 infection[6]. Although sialic acid is not a nanoparticle itself, it can be assembled into scaffolds with the capability to encase viruses.

Nanocellulose is a universal binder due to its high surface area that creates intricate networks supported by oriented attachments that facilitate multiple interactions between the nanoparticles themselves, and with other similar sized particles, with high interfacial energy and Brownian motion[7]. Nanocelluloses are utilized in coatings, energy storage devices, binders of natural fibers and composites, wound dressings, bone and cartilage restoration, drug delivery systems, tissue engineering, and an ever-increasing number of biotechnology and biomedical applications[8]. Recent studies have shown the feasibility of developing nanocellulose-based antimicrobial materials[9,10]. Nanocelluloses can interact with a wide range of biological entities including eukaryotic cells, bacteria, and viruses, and the type of interaction can be engineered through chemical synthesis[11,12].

Cellulose nanoparticles result from the deconstruction of larger cellulose microfibers. Both nanocellulose synthesis strategies and the source of cellulose fibers influence their type and the properties of the resulting material[13]. Aqueous suspensions of nanocellulose form hydrogels whose stabilization is a consequence of electrostatic and steric repulsions[14]. Functionalized nanocellulose with half-ester sulfate groups hold negative charges that create repulsion among the suspended nanoparticles, resulting in stable, single-phase colloid suspensions with time-independent rheological properties[14,15]. The supramolecular interactions between sulfated nanocellulose particles and other particles within the nanoscopic scale (including viruses) rely on the formation of linkages or electrostatic interactions between cellulose's active groups and charged moieties in proteins such as those on the surface of microbes.

Nanoparticles are prone to combat SARS-CoV-2 through agglutination around viral particles, and these antiviral properties can be programmed based on varying functional requirements. A recent report on nanocellulose, as a delivery system for the antiviral agent curcumin, was effective against SARS-CoV-2[16]. Nevertheless, no previous investigation focused on the use of nanocellulose particles to bind viruses or to entrap cell receptors or anti SARS-CoV-2 specific antibodies to create molecular lures.

In this research, we utilized the gelification property of sulfated nanocellulose to nanoencapsulate viruses, namely SARS-CoV-2 and HIV-1, or to immobilize molecular lures specific for SARS-CoV-2, to inhibit viral transmission. The nanoencapsulation is due to anionic interactions between sulfate groups in modified cellulose particles and positive charges in the virus membrane and surrounding medium. This prevents the viruses from binding with their cell receptors, namely ACE2 for SARS-CoV-2[17] and CD4 and coreceptor (CCR5 or CXCR4) for HIV-1[18].

## Results

### ENC encapsulation of S protein prevented binding to ACE2 in ELISA tests

To determine whether ENC can encapsulate SARS-CoV-2 S protein thus preventing interaction with ACE2, and to establish an optimal working concentration of ENC, we used the quantitative SARS-CoV-2 neutralizing antibody ELISA with modifications. Spike protein labeled with horse radish peroxidase (HRP) was preincubated with ENC suspension prior to its addition in ELISA wells then combined with biotinylated ACE2 receptor, which attaches to avidin coated plate wells. HRP-labeled S protein that binds ACE2 within the well was then measured after addition of HRP substrate. HRP enzymatic activity was directly proportional to the amount of S protein that escapes ENC. A diagram of the assay is depicted in Fig. 1a.

When S protein was preincubated in 0.27% w/v ENC before addition to the assay, the absorbance units (AU) obtained were below the limit of detection (0.142 ± 0.008 AU compared to lowest standard measurement of 0.291 ± 0.053 AU, n = 3). This indicated that S protein was irreversibly bound by ENC and was not able to react with ACE2. Validity of the assay was confirmed by use of two positive controls containing anti-SARS-CoV-2 S IgG provided with the kit and each gave measurements in the expected range provided with the assay instructions.

To determine the optimal ENC concentration for nanoencapsulating S protein, one experiment was run using a series of dilutions of ENC (0.5, 0.4, 0.33, 0.27, 0.16, and 0.1)% w/v with the addition of 0.9% w/v NaCl solution. The lower the absorbance, the more effective the S protein nanoencapsulation. According to the absorbance curve, we determined that 0.27% w/v ENC suspension was the optimal concentration (Fig. 1b).

### Afm And Fluorescence Confocal Microscopy Demonstrated Encapsulation Of S Protein In Enc

To demonstrate at the nanoscopic scale the interactions and crosslinking of ENC particles around the S protein clumps we used atomic force microscopy (AFM). This technique revealed intricate networks of ENC that were denser (bright spots) when attached to S protein clumps (Fig. 2a). This is due to a higher affinity of ENC to protein clumps than to other ENC particles caused by anionic interactions between the cellulose nanoparticles and protein positive moieties.

Confocal microscopy was employed to demonstrate at the micro scale the agglutination of S protein in ENC matrix, and to verify the identity of the presumed S protein clumps seen by AFM. The protein interaction with ENC was evident even after a series of washing cycles, suggesting the nanoencapsulation of protein clumps is irreversible. To demonstrate that, hydrogels encapsulating Alexa Fluor 488 (AF488) labeled S protein were crosslinked, washed in triplicate, and dried followed by confocal microscopy analysis. Figure 2b shows the same image with different filters: (i) confocal differential interference contrast image of the ENC membrane, (ii) AF488 confocal images of fluorescent clumps of S protein and (iii) overlay of DIC and AF488 images showing the fluorescent clumps of S protein on the ENC membrane.

## Sars-cov-2 S Pseudovirus Cell Entry Was Inhibited By Enc

To test whether ENC can prevent virus transmission, 293T-ACE2 cells were inoculated with SARS-CoV-2 pseudovirus (pv) with a green fluorescent protein (GFP) reporter in conditions with and without ENC and analyzed by flow cytometry three days later.

SARS-CoV-2 pv entrapped in ENC or captured by recombinant ACE2 protein (ACE2) or anti-SARS-CoV-2 S IgG (IgG) entrapped in ENC was not able to infect cells: 0.003 ± 0.003%, 0.007 ± 0.004%, and 0.022 ± 0.010% of infected cells respectively ($p < 0.0001$), ($n = 16$, Fig. 3a and dot plots in **Suppl. Figure 1**). Meanwhile, for controls in which SARS-CoV-2 pv was added without ENC, the number of cells infected was 1.76 ± 0.08% ($n = 16$). Preincubation of SARS-CoV-2 pv with either ACE2 or IgG did not significantly change virus transmission (1.94 ± 0.19% and 1.28 ± 0.10% infected cells, respectively, Fig. 3a). The low level of infection with SARS-CoV-2 pv entrapped by ENC or captured by ACE2 or IgG in ENC was not different than background in uninfected controls ($n = 9$, $p = 0.388–0.993$). These results were confirmed by fluorescent microscopy as well, where a similar pattern of GFP + cells was observed (**Suppl. Figure 2a-f**).

We next evaluated whether cells could be protected from infection by entrapping the cells in ENC. Cells were first embedded in ENC and then inoculated with SARS-CoV-2 pv in culture medium or SARS-CoV-2 pv in ENC. SARS-CoV-2 pv infection was evaluated by fluorescent microscopy. Cells were cultured as above then the gels were transferred to microscope slides at day 3 post-inoculation, stained with propidium iodide, cover-slipped and imaged on the microscope. All cells were visualized by CellTrace™ Violet dye (blue), dead cells were visualized by propidium iodide (red) staining, and infected cells were GFP positive (green). Positive control cells (Fig. 3b**)** were viable (few red cells) and demonstrated obvious signs of infection (green cells); whereas cells embedded in ENC (Fig. 3c,d**)** were viable (few red cells) but showed no signs of infection (no green cells)($n = 4$).

## Long-term Direct Cell Exposure To Enc Had A Transitory Effect On Cell Viability And Proliferation

To confirm that cells were viable, and that lack of infection in ENC conditions was not due to massive cell death, we evaluated cell viability in the above experiments by flow cytometry using AlexaFluor350 dye, which is taken up by dead cells. This analysis revealed small but significant decreases in cell viability in ENC conditions. The percentage of viable cells in positive controls (cells + SARS-CoV-2 pv) was 98.29 ± 0.23% (n = 16, Fig. 4a) and cells in ACE2 + SARS-CoV-2 pv, IgG + SARS-CoV-2 pv, and ENC + IgG + SARS-CoV-2 pv conditions were similar with 96.76 ± 0.88%, 98.11 ± 0.31% and 94.08 ± 1.49% viable cells, respectively. Cells in ENC + SARS-CoV-2 pv and ENC + ACE2 + SARS-CoV-2 pv conditions were statistically different with 94.41 ± 1.12% (p = 0.010), 92.44 ± 1.66% (p = 0.006) viable cells, respectively.

To further examine the effect of ENC on cells, CellTrace™ Violet dye dilution was used to assess proliferation index (PI) of cells and a pattern similar to viability data was observed. Positive control cells had a PI of 1.92 ± 0.14 (n = 15, Fig. 4b) and cells in ACE2 and IgG with SARS-CoV-2 pv conditions were not significantly different with PIs of 1.87 ± 0.12 and 2.03 ± 0.16 respectively. PIs for cells in ENC + SARS-CoV-2 pv, ENC + ACE2 + SARS-CoV-2 pv, and ENC + IgG + SARS-CoV-2 pv conditions were lower: 1.66 ± 0.10% (p = 0.008), 1.72 ± 0.10% (p = 0.101) and 1.57 ± 0.09% (p = 0.015) respectively. Cell infection by SARS-CoV-2 pv did not affect PI: infected and uninfected cells had PIs of 1.98 ± 0.11% and 2.00 ± 0.11% (n = 22, p = 0.813, Fig. 4c) respectively.

However, the effect of ENC on cell proliferation was transitory: experiments were performed in which cells were removed from all conditions after 4 hours or overnight incubation, washed and returned to fresh plates without re-addition of SARS-CoV-2 pv, ENC, ACE2, and IgG. PI was evaluated 3 days post-inoculation. Results show that when ENC is removed, cells recovered and PIs of all transferred cells were no longer significantly different in their proliferation rate from control cells (n = 3, p = 0.264–0.999, Fig. 5a,b).

## Enc Prevented Transmission Of Hiv-1 Into Target Cells

To test whether ENC prevented infection of other viruses, we next assessed its ability to entrap HIV-$1_{LAI.04}$. We infected MT-4 cells with HIV-$1_{LAI.04}$ in the presence of ENC. HIV-1 was diluted in NaCl and added either to culture medium or to ENC, and then applied to MT-4 cells for 4 hours, washed three times in PBS, and returned to culture. HIV-1 infection was evaluated 4 days post-infection by flow cytometry measurement of p24 + cells. In control experiments without ENC, HIV-1 infected 27.27 ± 2.62% of cells (n = 16, Fig. 6). In contrast, 0.05 ± 0.01% of cells were infected when HIV-1 was entrapped in ENC which was significantly less than in the controls (p < 0.0001) and not significantly above background staining of uninfected cells (0.04 ± 0.01% infected cells, p = 0.704).

### The Ability Of Enc To Completely Entrap Sars-cov-2 Pv Was Mediated By Sodium Chloride

We next examined the mechanism of ENC virus entrapment. It is known that sulfonation of nanocellulose results in ionic functionalization of cellulose nanoparticles that allow crosslinking mediated by positively charged ions such as sodium and calcium. We therefore tested whether this was indeed the mechanism by which virus was encapsulated within ENC. Conditions for SARS-CoV-2 pv infection were modified such that NaCl solutions were replaced with culture medium in the above experiments. In conditions without ENC, we observed infected cells similar to the above-mentioned experiments: 2.31 ± 0.34% and 2.74 ± 0.43% and in cells + SARS-CoV-2 pv, cells+(ACE2 + SARS-CoV-2 pv), respectively; however, the condition of cells+(IgG + SARS-CoV-2 pv) conditions did have lower infection: 1.55 ± 0.26% infected cells, ($p = 0.009$, $n = 11$, Fig. 7). Cells in ENC conditions showed significant reductions in SARS-CoV-2 pv infection compared to control infected cells (cells + SARS-CoV-2 pv): 1.37 ± 0.28% ($p = 0.007$ ), 1.43 ± 0.26% ($p = 0.024$) and 0.79 ± 0.17% ($p = 0.002$) infected cells for cells+(ENC + SARS-CoV-2 pv), cells+(ENC + ACE2 + SARS-CoV-2 pv) and cells+(ENC + IgG + SARS-CoV-2 pv) conditions respectively. However, the percentage of infected cells was higher than in the above experiments where NaCl was used for crosslinking the ENC and infection was almost completely suppressed.

## Discussion

The current study reports on a nanocellulose that is capable of efficiently preventing viral infection. Nanocellulose derived from ivory nut endosperm yields thin nanoparticles (from 1–5 nm width) which provide high surface area for entrapment of microbes. Here we demonstrate ENC as a universal binder that prevents viral infection of cells. ENC was shown to nanoencapsulate S protein of SARS-CoV-2, as well as whole SARS-CoV-2 pv and HIV-1 virions. Moreover, ENC demonstrated the ability to immobilize IgG and ACE2 recombinant protein that were utilized with high specificity as molecular lures to bind and immobilize SARS-CoV-2 pv.

ENC suspensions, when crosslinked, form hydrogels that result from the entanglement of nanoparticles mediated by electrostatic stabilization associated with positively charged molecules such as sodium, calcium and potassium ions. This crosslinking causes sol-to-gel transitions that are irreversible. The ENC particle aggregation that occurs during sol-to-gel transitions is a phenomenon ubiquitous in colloidal and aerosol systems, including nanocellulose aqueous suspensions. Upon dispersion, particles collide and often irreversibly stick together to form larger clusters in a fractal-like morphology[19]. The crosslink density of the gels is dependent on the concentration of nanoparticles, while the pore size of the matrix is an inverse function of the concentration[20]. Different chemical routes of surface functionalization of the nanocellulose can provide ionic surfaces via phosphorylation, carboxymethylation, oxidation, and sulfonation, or promote hydrophobic interactions through acetylation, etherification, silylation, urethanization, and amidation[21]. Here we used sulfonated ENC which allows electrostatic interactions between the cellulose's active groups and charged moieties in proteins such as those on the surface of viruses.

The feasibility of ENC as an inhibitor of viral transmission was first verified by ELISA, which demonstrated effective blocking of SARS-CoV-2 S protein interaction with ACE2 in the presence of ENC, and allowed us to identify optimal working concentrations of ENC. AFM and confocal microscopy validated these results, showing entrapment of S protein in ENC.

Results from SARS-CoV-2 pv infection of 293T-ACE2 cells demonstrated that ENC essentially eliminated infection, as no or few cells were infected. This was true whether SARS-CoV-2 pv was directly entrapped in ENC, or whether molecular lures (ACE2 or anti-SARS-CoV-2 IgG) were first entrapped and then allowed to bind SARS-CoV-2 pv. ACE2 and anti-SARS-CoV-2 IgG added directly to the culture medium were not effective at preventing infection. It should be noted that the experiments were performed with conditions that maximize the chance of infection; therefore, the design did not allow for optimal inhibition with ACE2 or IgG, which would require a short incubation time between cells and virus followed by removal of virus.

Also, infection was prevented when cells were embedded in ENC. This also points to the universal binding of ENC, as it can entrap not only microorganisms, but also eukaryotic cells. This expands the possibilities for other applications of ENC.

Exposure of cells to ENC for three days resulted in small decreases in cell viability and of proliferation indexes. However, these effects were transient as demonstrated by cell transfer assays. If cells were removed from ENC conditions after 4 hours or overnight incubation with ENC, their proliferation indexes returned to normal. Although we did not address the mechanism for these decreases, we believe it may be related to insufficient gas or nutrient exchange from the relatively thick ENC gels sitting on top of the cells. In future applications of this technology, it is unlikely that cells will be directly exposed to thick layers of ENC for long periods of time.

The ability of ENC to prevent viral transmission was not unique to SARS-CoV-2, as experiments with infectious HIV-1 demonstrated a similar effect. HIV-1 infection was almost completely suppressed when virus was entrapped in ENC. Thus, with a second virus, we showed the ability to block infection of cells by entrapping virus in ENC.

Taken altogether, these results demonstrate wide potential applicability of ENC that can bind individual proteins (S protein, ACE2, IgG), different virus particles (SARS-CoV-2 pv and HIV-1) and whole cells. The electrostatic interactions between the functionalized ENC and proteins form irreversible crosslinked hydrogels. To demonstrate the mechanism of entrapment by ENC, we established the importance of positively charged molecules, in this case NaCl, in crosslinking the ENC to permanently entrap particles. When NaCl was omitted from ENC preparations, some viral particles were able to escape the ENC scaffold and infect cells.

Our study does have several limitations. We assessed only a few proteins and viruses, and entrapment was assessed only over a relatively short period of time. Also, for future applications, more elaborate characterization of ENC will be required. Potential risks associated with the use of nanoparticles are a concern for bioaccumulation and other potential subcellular interactions. For instance, *in vivo* experiments with respirable nanocellulose and cellulose dust, after one month of exposure, resulted in granulomatous inflammation, fibrosis, and albeobronchiolitis[22]. Nevertheless, the ENC particles utilized in this work, due to their dense negative charge provided by $SO_3^-$ groups from sulfuric acid synthesis, have the capacity of instantaneous crosslinking in the presence of positive ions and molecules, changing from nanostructured materials to macrostructured scaffolds which are unable to penetrate cells. Rather, they can embed cells, viruses, and molecules. ENC particles act as building blocks that are assembled together into large crosslinked structures.

ENC is particularly well suited as a universal binder due to its high surface area for entrapment. The ability of ENC to encapsulate viral particles thus preventing their ability to infect target cells, as well as to also entrap proteins and cells, demonstrate the usefulness of this biomaterial for development of strategies to prevent viral transmission. This material has potential applications as a film for masks and filters, to bind viral particles increasing the effectiveness of these filtration devices, or to create adsorbent surfaces to capture or inactivate viruses. The concentrations of ENC used in these experiments will lend themselves easily to aerosilization making them ideal for such purposes. Nanoparticles that are deemed safe for *in vivo* use also have potential use as agents to enhance natural compounds by improving their bioavailablility, or for drug delivery purposes.

### Materials And Methods

## Nanocellulose synthesis

ENC was synthesized from ivory nut endosperm by grinding Tagua seeds to a particle size of < 200 µm then subjecting the flour (20 g) to acid hydrolysis in 8M sulfuric acid at 1:10 ratio (grams of flour: grams of sulfuric acid) at 60°C for 2.5 hours at 300 rpm. The reaction was stopped with an equal volume of deionized water. The suspension was collected in a 50 ml tube, centrifuged at 3000 rpm for 10 minutes, supernatant discarded, and the pellet resuspended in deionized water and dialyzed for one week, until reaching pH 5.

## Adjustment of ENC w/v percentage

ENC percentage w/v in the hydrogel was determined on three samples of 10 ml of hydrogel dried at 80°C for 48 hours. The dried ENC was weighed to calculate % w/v then diluted to 0.25% w/v and stored at 4°C.

## Titration of $SO3^-$ groups

Conductimetric titration was used to measure $–SO_3H^-$ groups of ENC that result from acid hydrolysis according to previously published methods[23]. The sample was prepared as follows: 2.5 g of sample suspension containing 0.803% w/v ENC was suspended in water. The sample was diluted in 120 ml of 1 mM NaCl solution, and neutralized with NaOH (2 mM) added in 0.1 ml aliquots at 30 s intervals[24]. The sulfate group's content was determined after three subsequent measurements[25]. The average value of volume of NaOH and conductometry were taken in order to plot the titration curve. The ester sulfate content of the samples was calculated using the equation: $\mathbf{X} = \mathbf{C}_t \cdot V_1 / m$ where: **X** is the sulfate group's content in μmol/g; $\mathbf{C}_t$ is the concentration of the NaOH solution, in μmol/l; $V_1$ is the volume of the NaOH solution consumed at the first intersection point in litres, and m is the oven-dry weight of sample in grams.

## ELISA tests

Quantitative SARS-CoV-2 Neutralizing Antibody ELISA Kit (Epitope Diagnostics, San Diego, CA) was used with modifications where S protein was preincubated with ENC before mixing with ACE2 reagent. Briefly, duplicate calibrators, controls, and samples were set up with 25 µl per well with 50 µl of HRP labeled spike protein, 40 µl of ENC at different percentages (0.5; 0.4; 0.33; 0.27; 0.16; and 0.1) w/v, and 10 µl of 0.015M NaCl. 50 µl of biotinylated ACE2 was added, mixed and incubated at room temperature for 45 minutes in the dark. Wells were washed 5 times, 100 µl substrate was added, plate was incubated at room temperature for 20 minutes in the dark, 100 µl of stop solution was added, and plate was read within 10 minutes on microplate reader at 450 nm absorbance.

## Atomic Force Microscopy

20 µl of ENC was deposited on a clean glass slide and 5 µl of different concentrations of SARS-CoV-2 S protein were dropped on top. The mix was left to interact then dry in ambient conditions. Atomic force microscopy (AFM) imaging was performed in air on a Bioscope Catalyst AFM (Bruker-nano, Santa Barbara, CA), in the "tapping" mode, using silicon cantilevers with nominal stiffness of 2.8 N/m and resonance frequency of 75 kHz (FESP cantilevers by Bruker-nano). Acquired images were post-processed using the instrument software (Nanoscope Analysis, v.2.0, Bruker-nano).

## Fluorescence Confocal Microscopy

Images of dry membranes resulting from pre-washed and dried ENC hydrogels encapsulating fluorescent S protein were acquired with an Olympus FV3000RS laser scanning confocal microscope using Peltier-cooled GaAsP detectors. Detector emission was automatically optimized for AlexaFluor 488 using a spectral system comprised of a volume phase holographic spectral transmission grating and adjustable slit. Images were collected with 30X silicon immersion objective (UPLSAPO30XS; NA 1.05; Olympus Tokyo). Spots on the bottom of a 35 mm glass bottom dish were acquired using multi-area time-lapse (MATL). Image fields were stitched with Olympus Fluoview software (FV31S-SW, Tokyo, Japan). Pixels were sampled by the Nyquist criterion.

## Cell lines and pseudoviruses/viruses

293T-ACE2 cells (Integral Molecular, Philadelphia, PA), target cells for SARS-CoV-2 pv, were cultured in DMEM, 10% FBS, 50 mg/ml gentamicin, 2.5 µg/ml amphotericin B and 0.5 µg/ml puromycin. SARS-CoV-2

S protein (Wuhan-Hu-1:D614) pseudotyped lentiviral stocks (Virongy, Manassas, VA) contained green fluorescent protein (GFP) reporters and were quantified by p24 content.

MT-4 cells (ATCC, Manassas, VA), target cells for HIV-$1_{LAI.04}$, were cultured in RPMI 1640, 10% FBS, 50 µg/ml gentamicin and 2.5 mg/ml amphotericin B. $HIV_{LAI.04}$ viral stock (Virology Quality Assurance Laboratory, Rush University, Chicago, IL) was quantified by p24 content.

Cell experiments were performed with 0.25% ENC, and recombinant ACE2 receptor (ACE2) and anti-SARS-CoV-2 S IgG (IgG) from Quantitative SARS-Cov-2 Neutralizing Antibody ELISA kit diluted 1:10 in 0.9M NaCl.

## Pseudovirus entry assay

293T-ACE2 cells (4 x $10^4$/well) in 100 µl of culture medium were placed in 96-well plates. SARS-CoV-2 pv (pv) was diluted in 0.9M NaCl for 100 ng/ml final p24 concentration. Conditions were set up in 3–4 replicates: 1) cells + pv: 10 µl pv resuspended in 90 µl medium; 2) cells+(ENC + pv): 10 µl pv in 90 µl ENC; cells+(ACE2 + pv): 10 µl pv in 90 µl ACE2; 3) cells+(ENC + ACE2 + pv): 80 µl of ENC incubated with 10 µl ACE2 for 15 minutes, then 10 µl pv added; 4) cells+(anti-SARS-CoV-2 IgG + pv): 80 µl medium, 10 µl IgG and 10 µl pv; 5) cells+(ENC + IgG + pv): 80 µl ENC incubated with 10 µl IgG for 15 minutes then 10 µl pv added. Solutions were incubated for 30 minutes at room temperature, added to cells and incubated for three days at 37$^oC$, 5% $CO_2$. Pseudoviral entry was observed by fluorescent microscopy and flow cytometry. Identical experiments were performed except that NaCl in all solutions was replaced with culture medium.

Cells to be embedded in ENC were resuspensed in NaCl with 10 µl cells at 4 x $10^6$ cells/ml added to 90 µl ENC for each well, and pv was added either as free pv or ENC encapsulated pv (10 µl pv in NaCl in 90 µl of medium or ENC).

For transfer experiments, cells were harvested after 4 hours or overnight incubation by trypsinization with 0.25% Trypsin-EDTA and centrifugation at 400xg for 5 minutes. Cells were washed twice with PBS, centrifuged, and cultured for the remainder of the 3 days in new plates.

## HIV infection assay

MT-4 cells (5 x $10^4$/well) in 100 µl of medium were placed in 96-well plates. Cells were inoculated with 1 ng/ml of HIV-1 p24 content diluted in 0.9M NaCl. Conditions were set up in 3–4 replicate wells: cells + HIV: 10 µl HIV-1 in 90 µl medium; cells+(ENC + HIV): 10 µl HIV-1 in 90 µl ENC; and cells only: 10 µl NaCl in 90 µl medium. Solutions were incubated for 20 minutes, added to cells and cultured for 4 days.

## Fluorescence microscopy and flow cytometry

Pseudovirus infection was monitored by visualization of GFP positive cells after three days on a fluorescent microscope (Keyence BZ-X810, Itasca, IL) equipped with filter cubes for GFP, Texas Red and

DAPI. For cell embedding conditions, gels were transferred to microscope slides, stained with propidium iodide (Thermo Fisher, Waltham, MA, 1 µg/ml in PBS), coverslipped and imaged.

Following imaging, cells were harvested by trypsinization, washed and labeled with Alexa Fluor 350 dye (ThermoFisher, 1 µg/ml) for viability, and acquired on a Symphony A5 flow cytometer (BD Biosciences, Franklin Lakes, NJ). Flow cytometry analysis included size gating on FSC vs SSC plots, single cells on FSC-A vs FSC-H plots, live cells by viability dye exclusion with BUV450 vs SSC, and BB515 staining for percent of GFP + cells. Analysis was performed in FlowJo v10.8 (BD Biosciences).

For proliferation, cells were prelabeled with Celltrace™ Violet (ThermoFisher) by suspension in 1 ml of PBS with 1 µl of dye, incubated for 20 minutes at 37$^{o}$C, and washed, with an aliquot fixed immediately. Cells were analyzed by flow cytometry with gating as above, followed by BV421 histograms for the proliferation modeling tool in FlowJo. Proliferation index (PI) was based on dye loss and was calculated as the total number of divisions divided by the number of cells that went into division.

For determining HIV infection, cells were labeled with viability dye, fixed and permeabilized (Fix and Perm™ cell permeability kit, ThermoFisher), then stained with mouse anti-p24 (KD57-RD1, Beckman Coulter, Brea, CA), washed and fixed in 2% paraformaldehyde. Cells were acquired on flow cytometer and p24 + infected cells were identified.

### P24 measurement by Luminex

SARS-CoV-2 pseudotyped lentiviral stocks and HIV-$1_{LAI.04}$ were quantified using a p24 cytometric bead assay[26].

### Statistical evaluation

Data normality was tested using D'Agostino and Pearson test or Shapiro-Wilk test when n was small. Normal data were analyzed by one-way analysis of variance and presented as mean ± standard error of the mean (SEM). Data not normally distributed were analyzed by Kruskal-Wallis Rank test and presented as median with interquartile range. GraphPad Prism v9.2.0 (GraphPad Software, San Diego, CA) was used for statistics with $p < 0.05$ considered statistically significant.

## Declarations

#### Acknowledgements

This work was supported by NIH intramural grant numbers: ZIA HD000713 to RDF, ZIA EB000085 to EKD, and ZIA HD008968-05 to LM; and the NIH Intramural Targeted Anti-COVID (ITAC) funding program.

#### Author contributions

JC conceived the project, RDF and LM directed the research; JC, WF, and ED contributed to the acquisition of data, analysis; all authors analyzed data and contributed to writing the manuscript; and all authors reviewed the manuscript.

#### Data availability

All data generated or analyzed during this study are included in this published article (and its Supplementary Information files).

#### Competing interests statement

The authors declare no competing interests.

## Figures

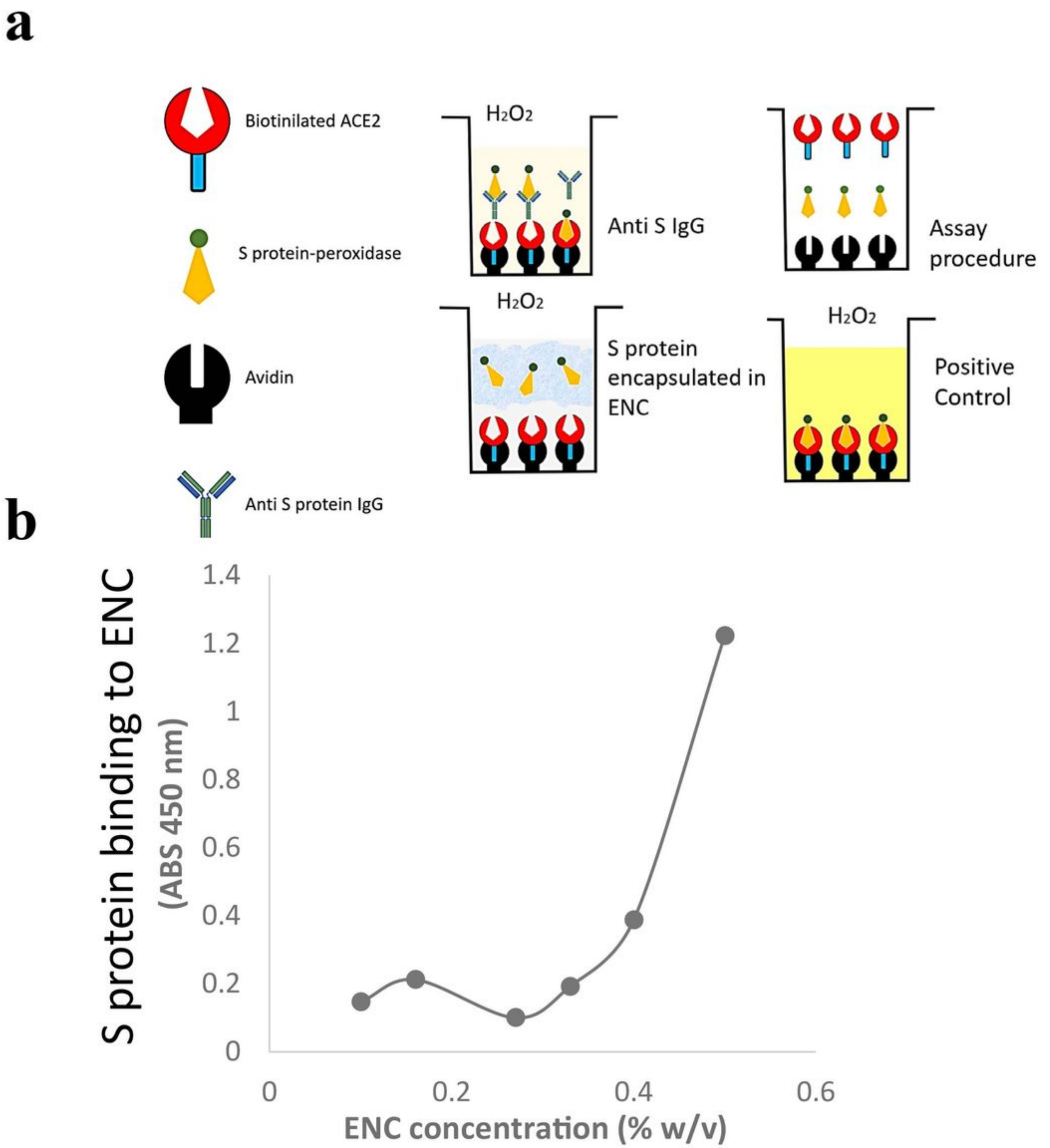

**Figure 1**

**ELISA experiments of S-protein encapsulation vs anti S IgG docking**

A modified ELISA protocol was used to demonstrate that SARS-CoV-2 S protein can be entrapped in endospermic nanocellulose (ENC) thus preventing its binding to ACE2. **a.** Schematic of the modified ELISA protocol for encapsulation of S protein. The assay was modified to measure the ability of ENC to block the interaction of S protein with ACE2, rather than the ability of anti-S neutralizing antibody to block this interaction; **b.**The modified ELISA was used to determine optimum % of ENC suspension (expressed in w/v) to efficiently encapsulate S protein; lower absorbance equals higher S protein encapsulation.

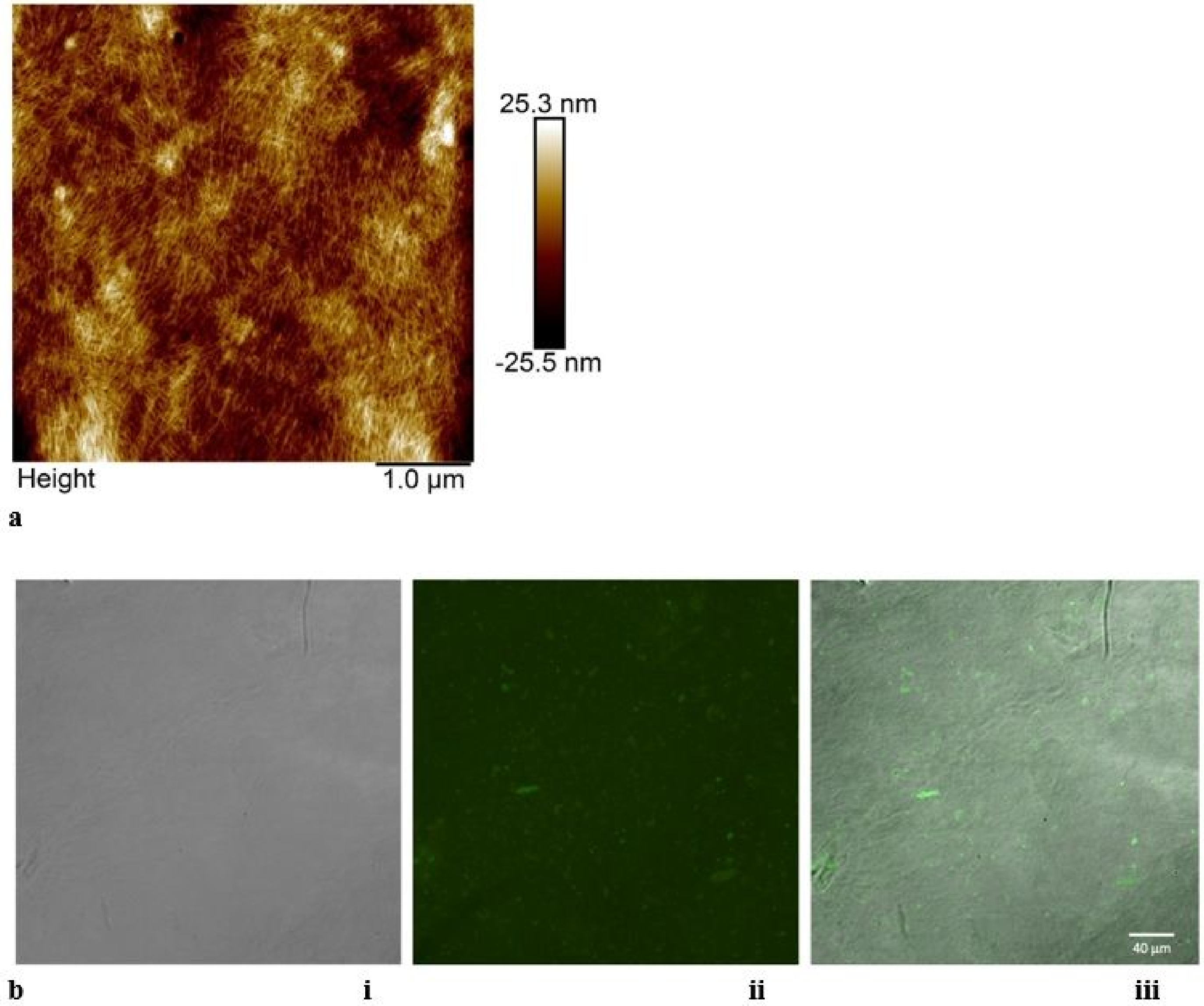

#### Figure 2

#### AFM and fluorescence images showed encapsulation of S protein

Atomic force microscopy image of encapsulated clumps of S protein via crosslinking of ENC. S protein was irreversibly encapsulated in endospermic nanocellulose (ENC) matrix due to crosslinking. Thin nanoparticles of 1-5 nm are seen in a crosslinked scaffold; **b**. Fluorescent S protein encapsulated in an ENC dried gel by differential interference contrast (DIC) imaging with fluorescent (488nm excitation) confocal microscopy: **(i)** DIC image of the ENC membrane; **(ii)**fluorescent confocal image of entrapped

Alexa Fluor 488 S protein clumps and **(iii)**overlay of ENC membrane showing the fluorescent clumps of S protein.

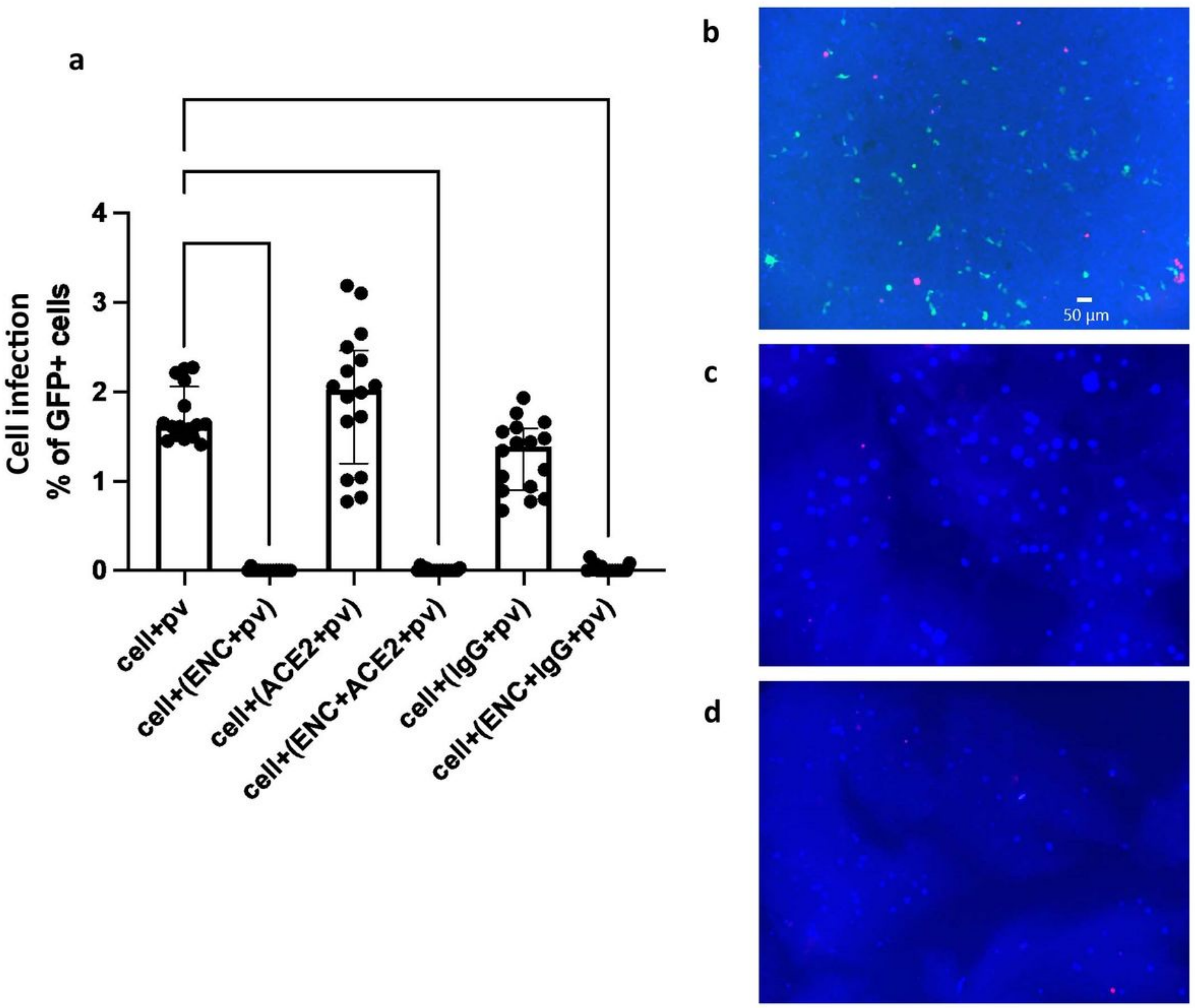

**Figure 3**

**ENC prevented SARS-CoV-2 pseudovirus infection of 293T-ACE2 cells.**

293T-ACE2 cells were inoculated with SARS-CoV-2 pseudovirus (pv) with a GFP reporter in conditions with and without endospermic nanocellulose (ENC) and molecular lures (ACE2 or anti-SARS-CoV-2 S IgG (IgG)) and incubated for 3 days. **a.** Cells were harvested for flow cytometry analysis and % of GFP infected cells was determined for each condition (n=16, ****$p<0.0001$, Kruskal-Wallis test, median with interquartile range). Fluorescent microscopy was used to image cells embedded in ENC and challenged with pseudovirus **(b-d)**; shown is one representative experiment out of four. **b.** infected cells in positive controls (cells+pv), **c.** cells embedded in ENC then infected with pv or **d.** cells embedded in ENC and infected with pv embedded in ENC. Blue represented all cells (CellTrace™ Violet), green indicated GFP infected cells, and Red indicated propidium idodide for dead cells. The ENC was visible as a faint blue cloud in **c** and **d** and demonstrates that cells are immobilized in the gel.

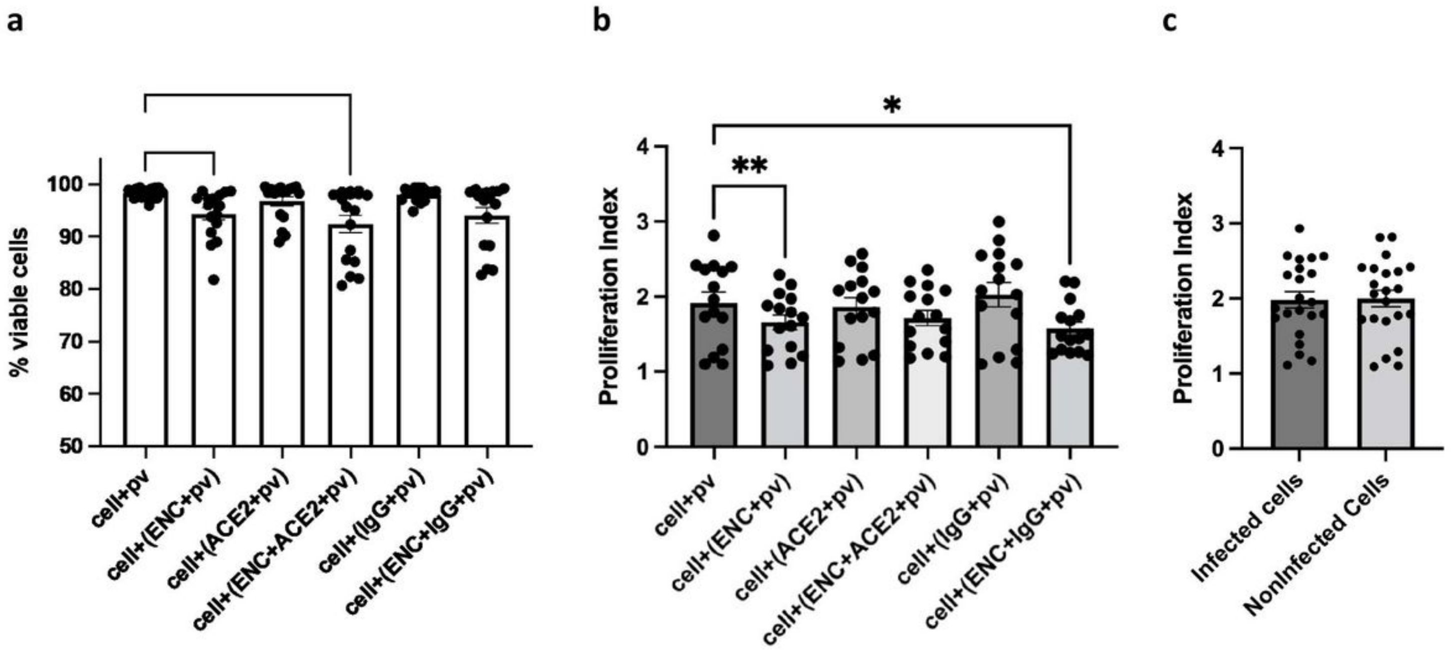

### Figure 4

**Viability and proliferation index was lower for cells in ENC.**

293T-ACE2 cells were inoculated with SARS-CoV-2 pseudovirus (pv) in conditions with and without endospermic nanocellulose (ENC) and molecular lures (ACE2 or anti-SARS-CoV-2 S IgG (IgG)) and incubated for 3 days. Cells were harvested for flow cytometry analysis. **a.** % of viable cells was calculated for each condition (n=16, **p<0.01, Kruskal-Wallis test, median with interquartile range). **b.** Proliferation index was measured by CellTrace Violet™ dye dilution for all conditions (n=15, *p<0.05, **p<0.01, ANOVA, mean ± SEM) and **c.** Proliferation index was compared between infected and noninfected cells in positive controls (cells+pv)(n=22, p=0.812, ANOVA, mean ± SEM).

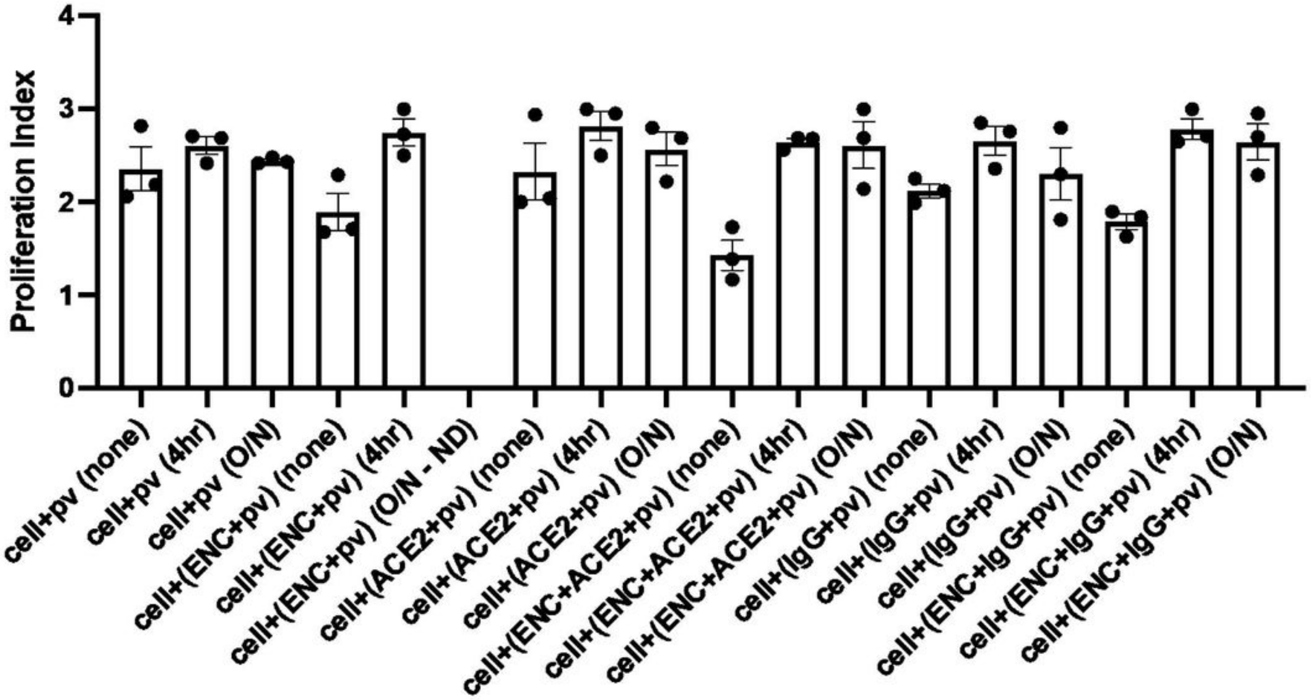

a b

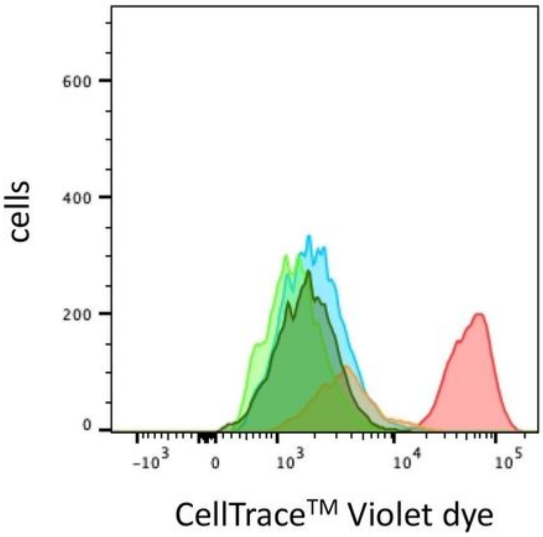

**Figure 5**

**Proliferation index recovered when ENC was removed.**

293T-ACE2 cells were inoculated with SARS-CoV-2 pseudovirus (pv) in conditions with and without endospermic nanocellulose (ENC) and molecular lures (ACE2 or anti-SARS-CoV-2 S IgG (IgG)) and incubated for 3 days (none) or cells were removed from ENC conditions after 4 hours (4hr) or overnight (O/N) and cultured in new plates. Cells were harvested for flow cytometry analysis and proliferation index was measured by CellTrace Violet™ dye dilution for all conditions. **a.** Proliferation of cells in transfer conditions was not significantly different from positive controls (cells+pv)(n=3, p=0.264-0.999, ANOVA, mean ± SEM). **b.** Histogram of CellTrace Violet™ dye dilution for initial staining intensity (red) and for cells+pv (blue), cells+(ENC+pv) no transfer (orange), cells+(ENC+pv) 4 hour transfer (light green) and cells+(ENC+pv) overnight transfer (dark green). Shown is one representative experiment out of three. ND, not done.

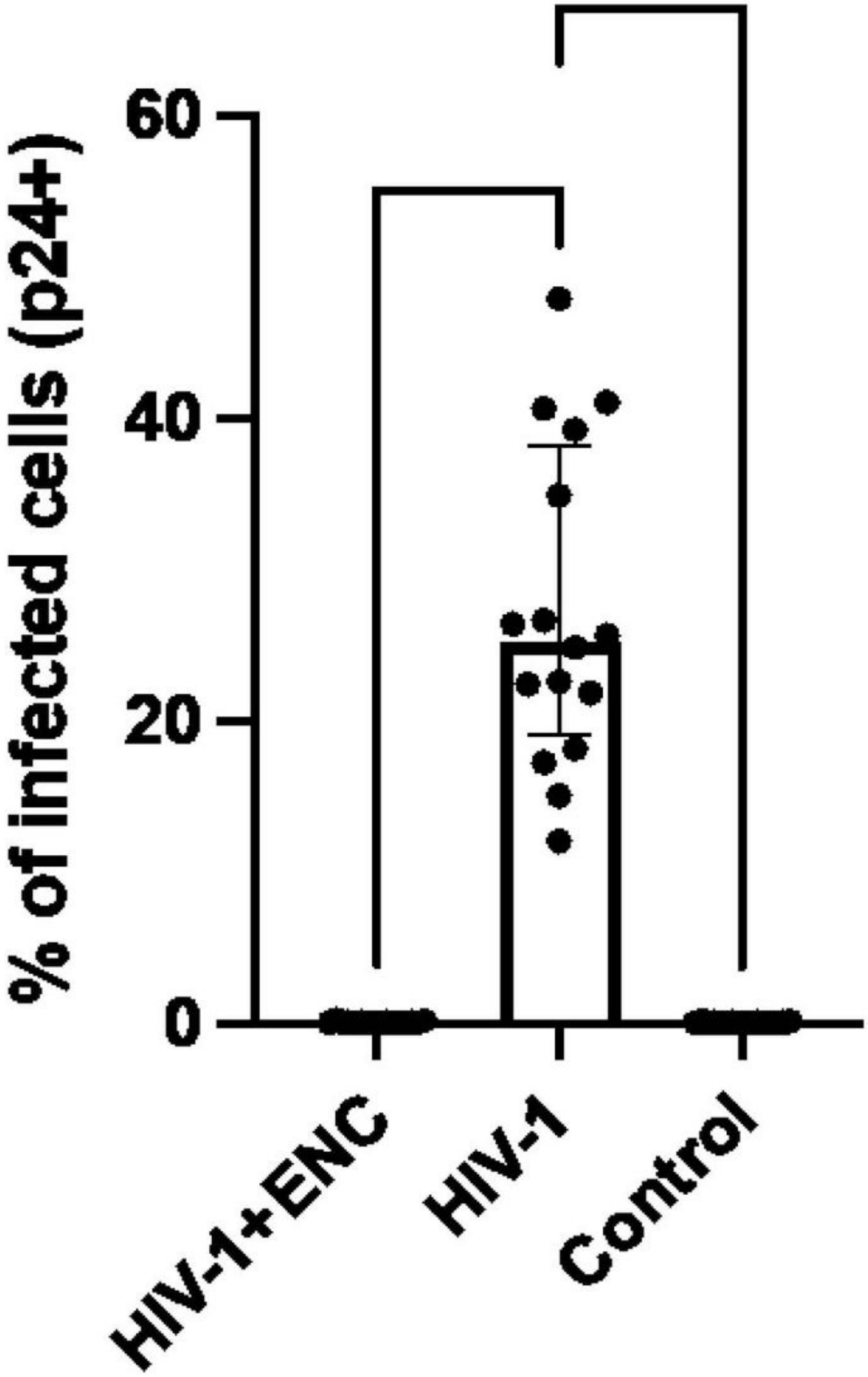

Figure 6

**ENC prevented HIV-1 infection of MT-4 cells.**

MT-4 cells were inoculated with HIV-$1_{LAI.04}$ in conditions with and without endospermic nanocellulose (ENC) for four hours, thoroughly washed and cultured for four days. Cells were harvested for flow cytometry analysis and infection measured by intracellular p24 staining. No infection of cells was observed in cells when virus was entrapped in ENC (n=16, ****p<0.0001 compared to positive control cells, Kruskal-Wallis test, median with interquartile range). The level of intracellular p24 in HIV-1+ENC conditions was not different from negative control cells with no HIV-1.

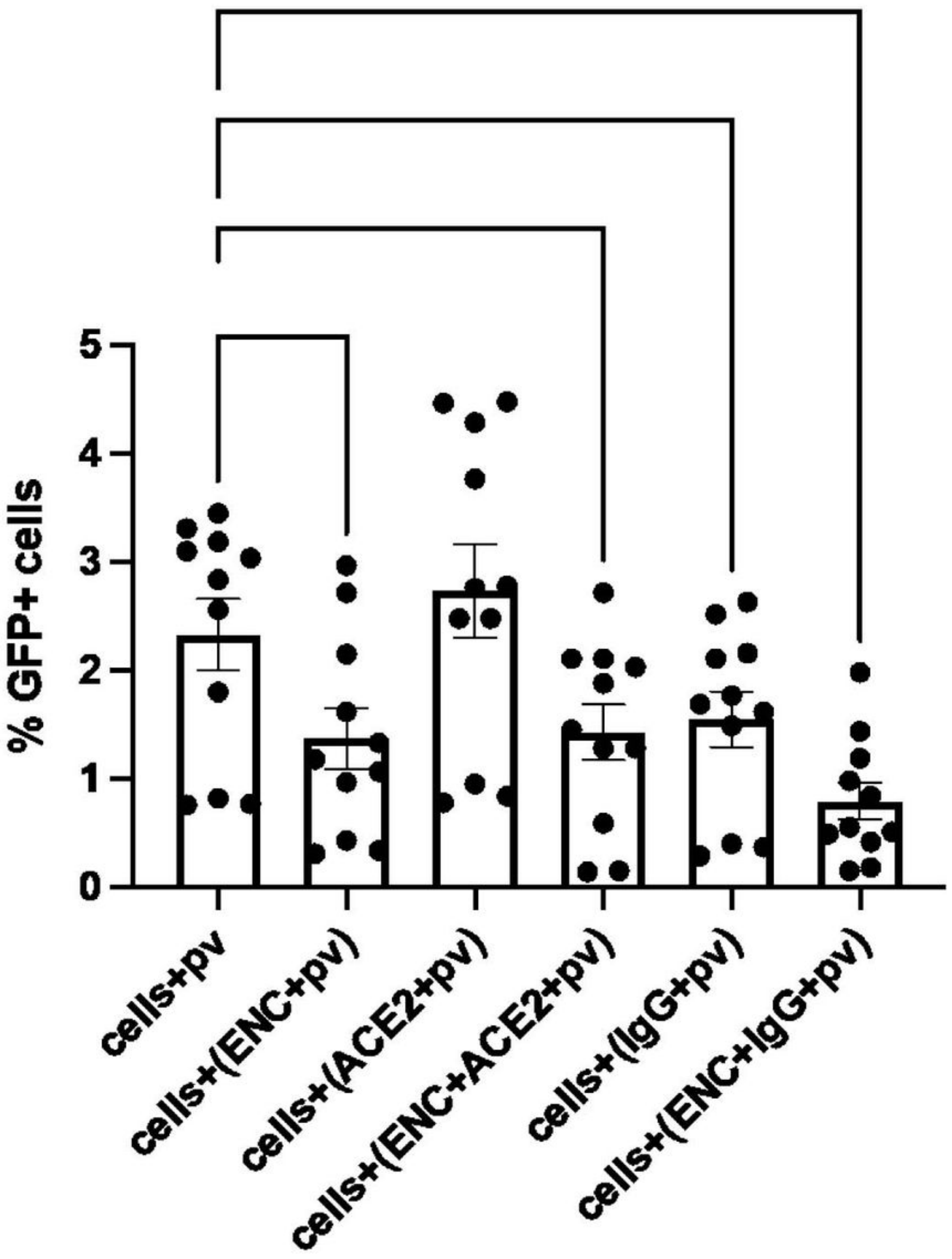

### Figure 7

**NaCl was critical for crosslinking and capture of virus in ENC.**

293T-ACE2 cells were inoculated with SARS-CoV-2 pseudovirus (pv) with a GFP reporter in conditions with and without endospermic nanocellulose (ENC) and molecular lures (ACE2 or anti-SARS-CoV-2 S IgG (IgG)) and incubated for 3 days, as in previous experiments except that NaCl was replaced with culture medium in preparation of reagents. Cells were harvested for flow cytometry analysis and % of GFP infected cells was displayed for each condition (n=16, *p<0.05, **p<0.01, ***p<0.001, ANOVA, mean ± SEM). ENC reduced but did not completely inhibit infection as in experiments where ENC was crosslinked by NaCl.

## Supplementary Files

This is a list of supplementary files associated with this preprint. Click to download.

- SARSCOV2NANOCELLULOSESupplement.docx
